# Controlling the Morphology of Poly(ethylene glycol)-b-poly(lactide) Self-Assemblies in Solution: Interplay of Homopolymer Additives and Kinetic Traps

**DOI:** 10.3390/nano14242015

**Published:** 2024-12-14

**Authors:** Pei Qi Lim, Srirangam Ramanujam Vaibavi, Atul N. Parikh, Subbu Venkatraman, Bertrand Czarny

**Affiliations:** 1 School of Materials Science & Engineering, Nanyang Technological University, Singapore 639798, Singapore; 2 Departments of Biomedical Engineering and Materials Science & Engineering, University of California, Davis, Davis, CA 95616, USA; 3 Material Science & Engineering, National University of Singapore, Singapore 117546, Singapore; 4 Lee Kong Chain School of Medicine, Nanyang Technological University, Singapore 636921, Singapore

**Keywords:** block copolymer, self-assembly, homopolymer, kinetics, morphology, micelle, vesicle

## Abstract

This study investigates the effects of homopolymer additives and kinetic traps on the self-assembly of poly(ethylene glycol)-b-poly(lactide) (PEG-PLA) block copolymer (BCP) nanostructures in aqueous environments. By using non-adsorbing PEG homopolymers to kinetically trap PEG-PLA nanostructures, we demonstrate that varying the concentration and molecular weight of the added PEG induces a reversible micelle-to-vesicle transition. This transition is primarily driven by changes in the molecular geometry of the PEG-PLA BCPs due to excluded volume screening effects. Additionally, the reversible vesicle-to-micelle transition upon PEG’s removal shows time and temperature dependency, highlighting the influence of the system’s kinetic nature. Intermediate structures observed during the transition support a mechanism based on shifts in the molecular geometry of PEG-PLA. As a proof of concept, we show that PEG-PLA vesicles can act as thermoresponsive delivery systems, retaining dye at low temperatures (4 °C) and releasing it upon heating (37 °C). Overall, this work presents a novel approach to controlling BCP nanostructures’ morphology, with implications for drug delivery and material science applications.

## 1. Introduction

Block copolymers (BCPs) are well known for their ability to self-assemble into a diverse array of nanostructures in solution [1,2], which makes them suitable for applications such as drug delivery [3], template synthesis [4], sensing [5], and catalysis [6]. The functions and properties of these nanostructures are closely linked to their morphology, and significant research has focused on the factors influencing the final self-assembled shape. In a selective solvent, BCPs’ morphology depends on the molecular geometry of the BCP molecule, determined by the balance between the collapsed solvophobic block (to reduce unfavorable solvent interactions) and the well-solvated solvophilic block [7]. The most direct approach to altering the molecular shape is by modifying the relative length of the polymer blocks, a method widely employed to tune self-assembled BCPs’ morphology [8,9,10]. With advancements in understanding BCP self-assembly, researchers are increasingly exploring how extrinsic and environmental factors like solvent conditions, temperature, and additives influence the final BCP assemblies [11,12,13]. Studies in this area have advanced our understanding of the complexity inherent in BCP self-assembly and paved the way for the creation of new classes of environmentally sensitive self-assembled structures.

Homopolymer additives present an intriguing parameter for influencing BCP self-assembly. Although homopolymers have been widely studied for their ability to modify BCP assembly in the bulk phase [14,15,16], fewer studies have examined their effects on BCP systems in solution [17]. A clearer understanding of homopolymers’ effects would allow for a broader utilization of this straightforward and yet powerful approach to manipulate BCPs’ morphology in solution, facilitating various applications. Additionally, this knowledge may offer insights into the behavior of self-assembled systems in complex, multicomponent settings.

Our interest lies in exploring how non-adsorbing homopolymer additives affect BCP self-assembled nanostructures in a solvent that is selective for the homopolymer and one of the BCP blocks. Previous studies on homopolymer and BCP micelle mixtures showed that the addition of homopolymers disrupts ordered BCP packing [18], leading to rheological changes and “melting” of micelle-based soft solids [19]. The addition of homopolymers has also been observed to induce macroscopic aggregation, causing gelation [20,21] and phase separation [22]. However, studies addressing how non-adsorbing homopolymers can induce morphological changes in dispersed BCP nanostructures are limited. Abbas et al. investigated homopolymers’ effects on hexagonally packed cylindrical micelles, revealing changes in hexagonal packing, order–order phase transitions, and phase separation with varying BCP and homopolymer compositions [23]. However, their focus was on long-range ordered phases at high BCP concentrations (>32% by volume), with morphological changes in dispersed BCP nanostructures at lower concentrations largely unexplored. Yang et al. used computational methods to show that repulsive homopolymers can transform discrete nanoparticles formed by AB diblock and BAB triblock copolymers in solution, though these effects remain to be verified experimentally [24]. In a more recent study, Garcia and co-workers examined the colloidal stability of micellar solutions in solutions containing the homopolymer. The authors used numerical lattice computations based on the self-consistent field theory (SCFT) and supported by dynamic light scattering observations of an experimental model. At both the adsorption and depletion limits, the authors found that the addition of homopolymers influences micelle–micelle interactions by affecting the corona’s thickness [22].

In the present study, we sought to address the existing knowledge gaps by investigating the effects of homopolymers on discrete BCP nanoparticles across a wider range of homopolymer concentrations through comprehensive experimental methods. We employed a system of poly(ethylene glycol)-b-poly(lactide) (PEG-PLA), a diblock copolymer, and poly(ethylene glycol) (PEG), a non-associating hydrophilic homopolymer, in water. This system was selected for its structural simplicity and availability, and the biocompatibility of PEG and the biodegradability of PLA, making it relevant for potential biomedical and consumer applications [25,26]. The PEG-PLA system also offers a unique advantage: The glassy nature of the PLA block at room temperature [27,28,29] allows us to kinetically trap BCP structures, enabling us to investigate the effects of high homopolymer concentrations using standard imaging and scattering techniques after the homopolymers’ removal.

In this study, we examined how PEG homopolymer additives and the kinetic traps provided by the PLA block enable controlled manipulation of PEG-PLA nanoparticle morphologies. We report how different concentrations and molecular weights (MWs) of PEG homopolymers drive the formation of various morphologies within PEG-PLA, facilitated by kinetic trapping of the BCP nanostructures. Additionally, we demonstrate the reversible vesicle-to-micelle transition upon homopolymer removal and the role of kinetic traps in governing this process. Finally, we investigated the potential of PEG-PLA vesicles as thermoresponsive delivery systems. This work highlights how careful consideration of BCP self-assembly and the surrounding conditions can yield novel material properties for practical applications.

## 2. Experimental Methods

### 2.1. Materials

PEG-PLA (Mn of PEG block = 5000 g/mol, Mn of PLA block = 10,000 g/mol) was obtained from Advanced Polymer Materials. The homopolymer PEG (MW: 2000 g/mol, 6000 g/mol, and 10,000 g/mol) was purchased from Sigma-Aldrich, Singapore; these homopolymers are referred to as PEG2k, PEG6k, and PEG10k, respectively. Additional chemicals such as acetone, NaFluo, and ammonium molybdate were obtained from Sigma-Aldrich, Singapore. All polymers and reagents used in the experiments were obtained from commercial suppliers and used without further purification.

Refer to the Supplementary File for material information on PVP, dextran, and the fabrication and characterization of BCPs, DLS, and DSC.

### 2.2. Fabrication of BCP Self-Assembled Structures

PEG-PLA was first dissolved in acetone. This solution was then pipetted into a 2 mL vial and left to dry, resulting in a 5 mg polymer film. Subsequently, the hydrating solution was added to the vial and the contents were subjected to 2 min of sonication for 2 cycles using a probe sonicator (Q125, QSONICA, Fischer Scientific Singapore) equipped with a 1/8” probe to promote self-assembly. Depending on the experiment, the hydrating solution could be deionized water or a pre-prepared aqueous solution containing the PEG homopolymers. In this paper, the concentration of PEG homopolymers is expressed in terms of the weight ratio between the added homopolymer and water. After sonication, the sample was allowed to cool to room temperature before being diluted with deionized water and subjected post-fabrication purification by dialysis.

### 2.3. TEM Characterization

The TEM grids were first treated with glow discharge using a hydrophilic treatment device (HDT 400, JEOL DATUM, NTU common TEM facility). Two microliters of the sample (which had been diluted to 0.25 mg/mL using deionized water) was pipetted onto the TEM grid. After 30 s, filter paper was used to wick off the excess sample solution. Two microliters of the negative stain (2 w/v% ammonium molybdate in deionized water) was then pipetted onto the sample and left to sit for 1 min. Filter paper was used to wick off the excess stain solution, and the sample was left to dry in air before imaging via TEM (Libra 120 Plus, Carl Zeiss) using a 2 k × 2 k CCD camera (Troendle (TRS), Sharp eye). Emsis, Germany.

### 2.4. Reverse Shape Transition

PEG-PLA vesicles were prepared as described above, using an aqueous solution containing 1 *w*/*w*% PEG6k. The vesicle suspension was then divided into 200 µL aliquots in Eppendorf tubes. These aliquots were stored at various temperatures (4 °C, 37 °C, and 60 °C). At designated time points (1 h and 24 h), the samples were removed from the ovens and immediately quenched in ice water to halt any shape transition. The samples were subsequently imaged using TEM under the previously described imaging conditions.

### 2.5. UV-Vis Characterization

A UV-Vis-NIR spectrophotometer (Cary 5000, Agilent Technologies, Singapore) was used to measure the samples’ absorbance at 600 nm at controlled temperatures (30 °C, 40 °C, and 50 °C), with absorbance serving as an indicator of nanoparticle size changes in solution. Samples were held in quartz cuvettes with deionized water as the reference. Temperature control was achieved using the Varian Cary Dual Cell Peltier accessory, and each sample was maintained at the target temperature for 30 min. Absorbance readings were recorded at 10 Hz, and all measurements were performed in triplicate.

### 2.6. Cargo Release from Vesicle Samples

For experiments involving cargo release from vesicle samples, 50 mg of PEG5k-PDLLA10k was hydrated with 1 mL of a 1 *w*/*w*% PEG6k aqueous solution containing 50 mg/mL of NaFluo. This sample was processed using the previously described fabrication method and purified via centrifugation. For the release experiment,1 mL of the dye loaded samples was sealed in 10 kDa regenerated cellulose dialysis bags (Spectrum Labs, Singapore). These dialysis bags were then placed in vials containing 10 mL of deionized water as the external buffer and stored at different temperatures (4 °C and 37 °C). At specific timepoints, a sample was taken from the external buffer before the entire buffer was replaced with a fresh 10 mL of deionized water. Using an independently prepared standard curve, the fluorescence intensity of the collected sample was measured to track the release of NaFluo. Fluorescence measurements were obtained with a microplate reader (Infinite 200, Tecan, Singapore) at an excitation wavelength of 480 nm and an emission wavelength of 520 nm.

### 2.7. Statistical Analysis

All experiments were performed in triplicate. Where applicable, the means and standard deviations were calculated, and Student’s *t*-test (with the *p*-value set at 0.05) was conducted to determine significant differences.

## 3. Results

### 3.1. Presence of Free Homopolymer Causes the Micelle-to-Vesicle Transition

**Figure 1:**
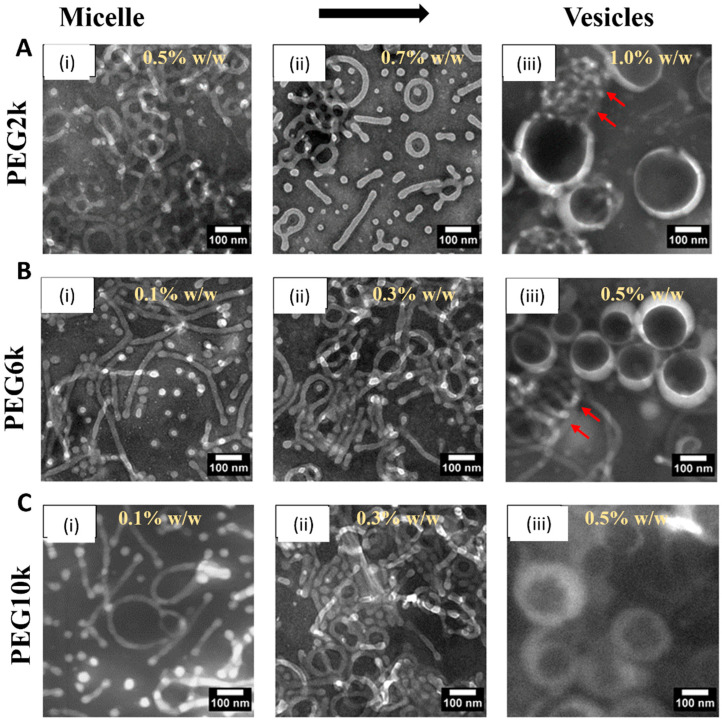
Transmission electron microscopy (TEM) images of PEG-PLA structures formed in deionized water with varying PEG molecular weights and concentrations (**A**) PEG2k dissolved in deionized water at concentrations of (i) 0.5% *w*/*w*, (ii) 0.7% *w*/*w*, and (iii) 1.0% *w*/*w*. (**B**) PEG6k dissolved in deionized water at concentrations of (i) 0.1% *w*/*w*, (ii) 0.3% *w*/*w*, and (iii) 0.5% *w*/*w*. (**C**) PEG10k dissolved in deionized water at concentrations of (i) 0.1% *w*/*w*, (ii) 0.3% *w*/*w*, and (iii) 0.5% *w*/*w*. Scale bars are indicated on individual panels.

Figure 1 and Figure S1 depict the morphological evolution of PEG-PLA nanostructures under varying PEG molecular weights and concentrations, as observed through TEM imaging. The results demonstrate a clear transition in the polymers’ morphology from micelles to vesicles as the PEG concentration increases (from (i) to (iii) in Figure 1). For PEG with a molecular weight of 2k (PEG2k), vesicle formation occurs only at a higher concentration of 1% *w*/*w*, and some micelles remain, as indicated by the red arrows. In contrast, for PEG6k, this micelle-to-vesicle transition is observed at a lower concentration of 0.5% *w*/*w*, although a few micelles still persist (red arrows). At the higher molecular weight studied (PEG10k), a complete transition from micelles to vesicles is observed at 0.5% *w*/*w*.

These observations highlight the critical roles of PEG molecular weight and concentration in determining the morphology of PEG-PLA assemblies. Increasing the molecular weight (PEG2k → PEG6k → PEG10k) enhances the hydrophilic chain’s ability to stabilize larger hydrophobic PLA cores, resulting in morphological transitions from micelles (PEG2k) to vesicles (PEG6k and PEG10k). Simultaneously, higher concentrations favor denser packing, which promotes transitions to more complex morphologies. Notably, PEG10k shows a lower critical concentration for vesicle formation, underscoring the impact of hydrophilic blocks’ length on reducing interfacial tension and achieving stable vesicles.

The underlying mechanisms driving these transitions include enhanced block copolymer hydration, where the hydrophilic PEG segment’s interaction with water increases with the molecular weight, stabilizing the assemblies. Longer PEG chains also enable greater flexibility and chain interpenetration, reducing the free energy in vesicular configurations. Additionally, the glassy nature of the PLA block may create kinetic traps that hinder equilibrium restructuring, resulting in kinetically trapped morphologies, depending on the preparation conditions. Overall, these results underscore the tunability of PEG-PLA nanostructures through controlled molecular weights and concentrations, offering valuable insights for designing functional nanomaterials. Such control is particularly important for applications like drug delivery, where specific morphologies, such as vesicles, are desirable for efficient encapsulation and release.

### 3.2. Shape Transition Is Reversible upon the Homopolymers’ Removal and Is Time- and Temperature-Dependent

**Figure 2:**
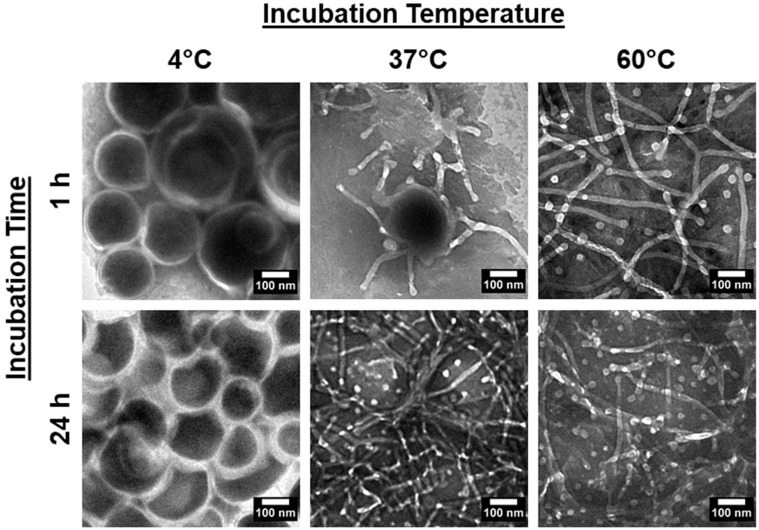
TEM images of PEG-PLA vesicles after incubation at 4 °C, 37 °C, and 60 °C for 1 h and 24 h, depicting the resulting structural changes under each condition.

Next, we considered if the kinetic nature of the system suggests time and temperature dependencies in the vesicle-to-micelle transitions. To validate this, PEG-PLA vesicles were incubated at 4 °C, 37 °C, and 60 °C for 1 h and 24 h, and their structures were analyzed using TEM. The results are presented in Figure 2.

In the 4 °C samples, no significant changes in the vesicular structure were observed, regardless of the incubation time. However, in the 37 °C and 60 °C samples, morphological changes were evident, with a clear difference in the rate of transition between the two temperatures. After 24 h, both samples had fully reverted to micelles. At the 1 h mark, only the 60 °C sample had transitioned to micelles, while the 37 °C sample was still in the transition phase, displaying broken or flattened vesicles with frayed edges. These findings confirm that the shape transition is reversible and dependent on both time and temperature.

**Figure 3:**
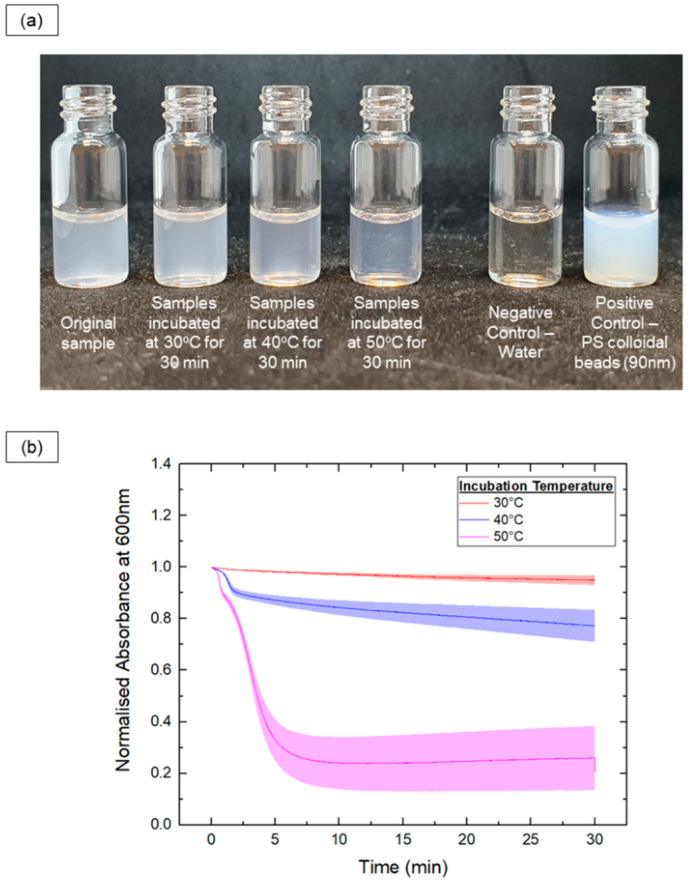
In situ monitoring of the samples’ turbidity upon incubation at various temperatures (30 °C, 40 °C and 50 °C) for 30 mins. (**a**) Photos showing the turbidity of PEG-PLA samples before and after the incubation process. (**b**) Plot of normalized absorbance at 600 nm (to track changes in turbidity) against time for each of the different samples.

Additionally, in situ optical methods were used to track the vesicle-to-micelle transition by measuring turbidity, which correlates with particle size. The absorbance at 600 nm was recorded for PEG-PLA vesicles heated at 30 °C, 40 °C, and 50 °C for 30 min, as shown in Figure 3.

In the 30 °C samples, there were no significant changes in turbidity, indicating little to no shape transition. In contrast, the 50 °C samples exhibited a rapid decrease in turbidity, with the absorbance reaching a plateau after 7 min, indicating a fast shape transition. The 40 °C samples showed a slower decrease in turbidity, suggesting a slower transition rate compared with the 50 °C samples. Further, a sharp drop in absorbance was observed early in the readings for the 40 °C and 50 °C samples, likely indicating a rapid initial decrease in size during the shape transition.

### 3.3. Transition Intermediates During Vesicle-to-Micelle Conversion and Their Relation to Packing Geometry

**Figure 4:**
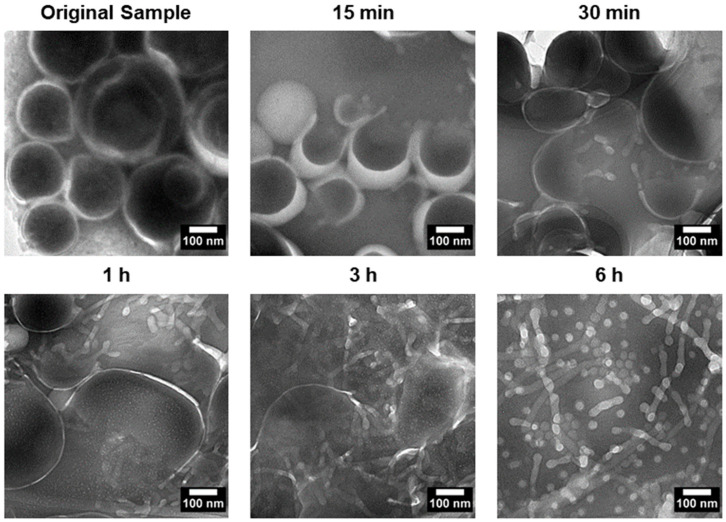
TEM images depicting the shape transition of PEG-PLA vesicles incubated at 37 °C over a 6 h period. The images show key intermediate stages of the transition, including porated vesicles at 15 min, a “jellyfish” structure at 30 min with an open bilayer and elongated edges, and irregular clumps with extended protrusions observed after 3 h. These intermediates illustrate the progressive transformation of vesicles into micelles, capturing the dynamic structural changes during the vesicle-to-micelle conversion.

To investigate the transition process, PEG-PLA vesicles were incubated at 37 °C to induce the shape transition. Aliquots were taken at specific time points, quenched in 4 °C water, and imaged using TEM. The results, presented in Figure 4, revealed distinct structural intermediates during the transition process. After 15 min, vesicles with pores were observed, followed by the appearance of a “jellyfish” structure at 30 min, characterized by an open bilayer with elongated edges. At 3 h, irregular clumps with extended protrusions were observed, marking a further stage in the transition. These observations suggest that the vesicle-to-micelle transition progresses through well-defined intermediate structures, potentially offering a strategy for generating novel morphologies.

### 3.4. Effect of PEG Homopolymers on PEG-PLA Self-Assembly

The self-assembly of PEG-PLA (PEG block Mn = 5000 g/mol, PLA block Mn = 10,000 g/mol) from a dried thin film was induced by probe sonication. This process helped to overcome the kinetic barrier caused by the glassy nature of the PLA block at room temperature. Once sonication was complete and the sample had cooled, the PEG-PLA returned to a kinetically trapped state. This provided an opportunity to remove the PEG homopolymers post-assembly without altering the pre-formed PEG-PLA structures.

**Figure 5:**
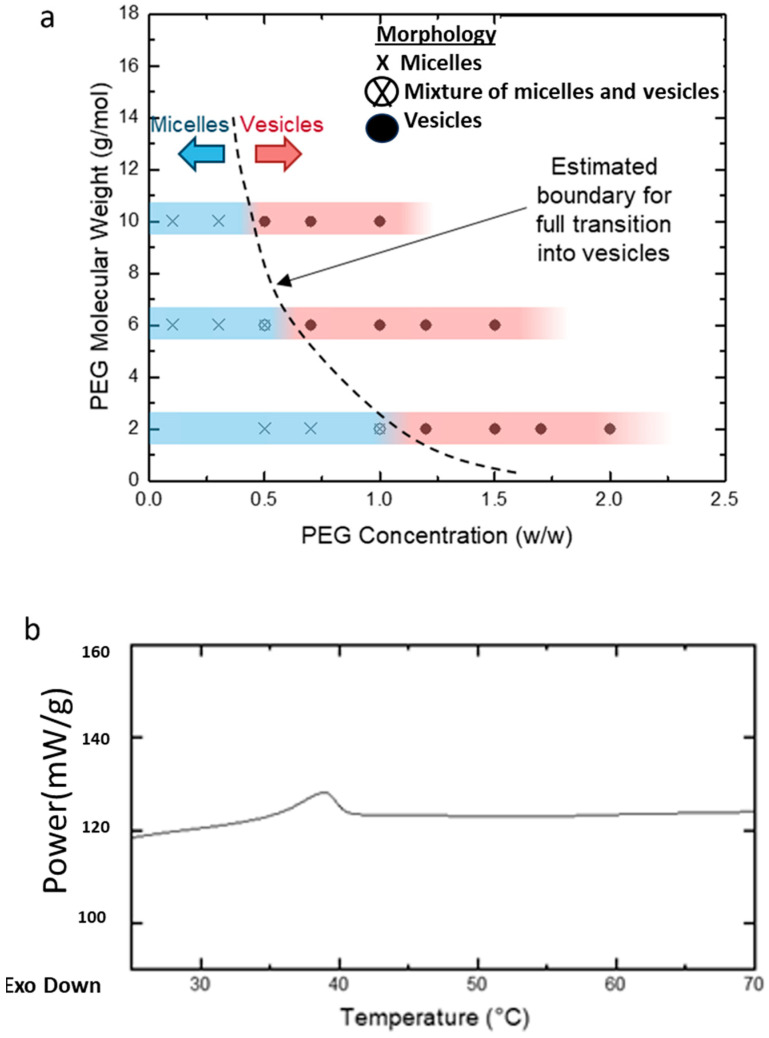
(**a**) Diagram illustrating the morphological transitions of PEG-PLA structures at varying PEG concentrations and molecular weights (MW), as determined by transmission electron microscopy (TEM). The blue regions on the bars indicate samples in which micelles (spherical or cylindrical) were observed, while the red regions highlight samples with vesicle formation. The transition zone, where both micelles and vesicles coexist, is represented between the blue and red areas. The dotted line marks the estimated boundary of this transition across different PEG concentrations and molecular weights. (**b**) DSC analysis of PEG-PLA structures formed in deionized water.

Various concentrations and MWs of PEG homopolymers (PEG2k, PEG6k, and PEG10k, as mentioned in Figure 1) were studied. The homopolymer concentration was expressed as a weight ratio between the added PEG and water. Transmission electron microscopy (TEM) revealed two main effects of the addition of homopolymers on the PEG-PLA self-assembly. The first was a concentration-dependent micelle-to-vesicle shape transition was observed, regardless of the MW of PEG. At low concentrations of PEG, spherical and cylindrical micelles formed. Increasing PEG concentrations promoted the formation of vesicles. Second, higher MW PEG triggered this shape transition at lower concentrations. For example, 1.2 *w*/*w* of PEG2k was needed to induce the transition, while only 0.7 *w*/*w* of PEG10k sufficed to cause a similar effect (Figure 5a).

To investigate the kinetically trapped nature of PEG-PLA structures, we hypothesized that the glassy behavior of the PLA block at room temperature stabilizes these nanostructures in a non-equilibrium conformation. Differential scanning calorimetry (DSC) analysis was conducted on PEG-PLA structures formed in water (Figure 5b). The DSC results revealed a glass transition temperature (Tg) of approximately 32 °C, marked by the midpoint of the stepwise transition in the heating profile. This Tg aligns with previously reported values, supporting the presence of a glassy state under ambient conditions. Additionally, a minor endothermic peak observed following the Tg likely corresponds to the relaxation enthalpy of the polymer.

### 3.5. Shape Transition in PEG-PLA BCPs Induced by PEG Homopolymers

**Figure 6:**
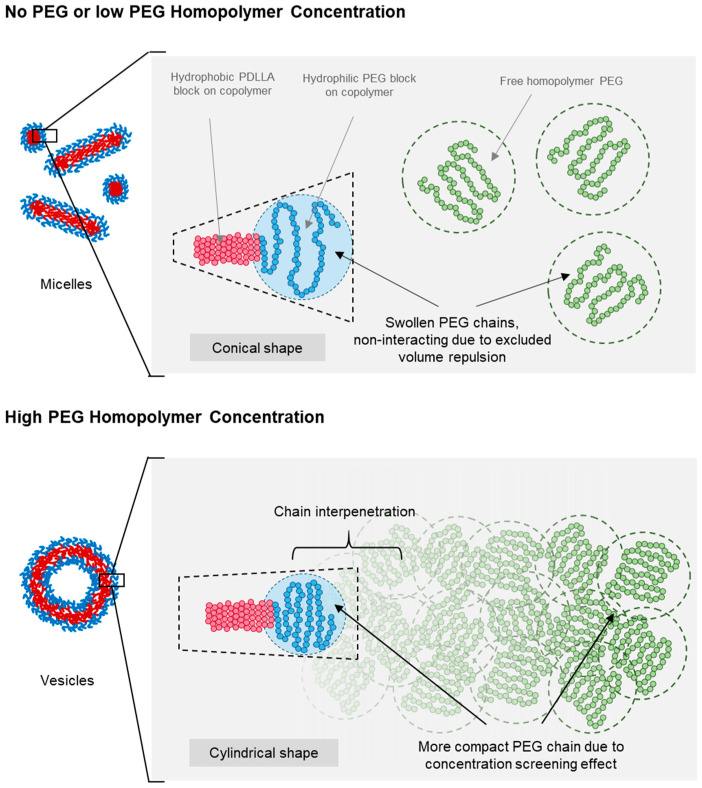
Diagram illustrating the proposed mechanism for shape transition driven by the screening of the excluded volume effect. The schematic shows changes in PEG chain size and BCP geometry with increasing PEG concentrations. Red represents the hydrophobic PLA block, blue represents the hydrophilic PEG block, and green represents the added free PEG homopolymers.

Figure 6 presents a schematic of the proposed micelle-to-vesicle shape transition mechanism in PEG-PLA BCPs induced by the addition of PEG homopolymers. When no or minimal PEG homopolymers were added, the solution remained diluted, and all PEG chains—both the added homopolymer and the hydrophilic block of PEG-PLA—existed as swollen and separate chains. This is attributed to excluded volume effects, which minimized interactions between chain segments and different PEG chains. As a result, the expanded conformation of the hydrophilic PEG head group in PEG-PLA led to a more conical packing geometry, favoring micelle formation at lower concentrations of PEG homopolymers.

At higher concentrations of PEG homopolymers, a combination of excluded volume screening, and interfacial and depletion effects contributed to the observed shape transition of PEG-PLA assemblies. This transition was dependent on the PEG concentration, occurring at lower concentrations with an increasing molecular weight of PEG. As the PEG concentration rose, the probability of chain overlap increased, reducing excluded volume interactions and leading to compaction of the polymer coils. This change resulted in a more cylindrical packing of the PEG head groups, favoring vesicle formation.

The transition to vesicles occurred at lower PEG concentrations for a higher molecular weight PEG, due to the larger radius of gyration in longer PEG chains, which facilitated earlier chain overlap. Additionally, the lower polarity of PEG compared with water enhanced interfacial effects that may have promoted planar lamellar structures, further supporting the transition to a vesicle morphology.

**Figure 7:**
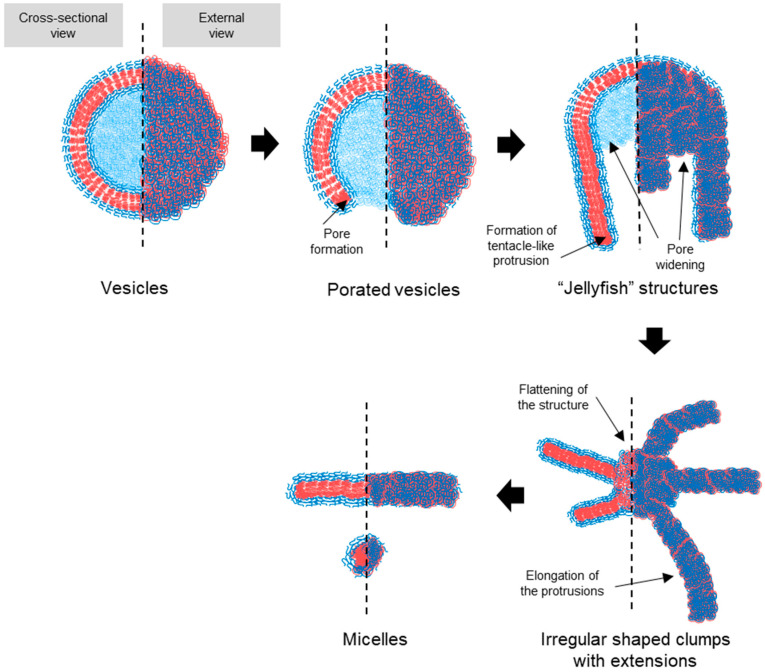
Schematic representation of the proposed pathway for the transition of PEG-PLA vesicles into micelles. The illustration details the sequential structural changes that occur during the transition process. The hydrophilic PEG block is represented by blue, while the hydrophobic PLA block of the BCPs is depicted in red. The pathway begins with pore formation in the vesicles, leading to a curved, open lamella structure. This structure evolves into a “jellyfish” configuration, characterized by an expanded pore and frayed edges that extend into elongated protrusions. As the process continues, these protrusions elongate further, and the bilayer structure recedes, ultimately resulting in the complete dissociation of the bilayer and stabilization of the micellar structures. This schematic highlights the relationship between the packing geometry of the BCPs and the observed morphological transitions.

Based on the TEM observations, a stepwise pathway for vesicle dissociation was proposed (illustrated in Figure 7). The process begins with pore formation on vesicles, leading to the emergence of a curved, open lamella structure. This then transforms into the “jellyfish” configuration, where the pore expands into an open bilayer, forming the “cap” of the jellyfish, while the frayed edges elongate into protrusions, representing the “tentacles”. As the transition continues, the protrusions extend further, and the bilayer flattens and recedes, resulting in irregular bilayer regions. Finally, these bilayer structures disappear entirely, leaving only micelles, the most stable form in water at room temperature. No additional transitions were observed beyond this final stage.

### 3.6. The Shape Transition Effect Was Not Observed with Other Non-Interacting Hydrophilic Homopolymers

Next, we tested whether the shape transition observed with PEG-PLA could be replicated using other non-interacting hydrophilic homopolymers. To investigate this, we induced the self-assembly of PEG-PLA in the presence of dextran and polyvinylpyrrolidone (PVP) homopolymers with molecular weights of 10,000 g/mol (referred to as Dextran10k and PVP10k, respectively) at various concentrations and compared the outcomes with those previously obtained with PEG10k.

**Figure 8:**
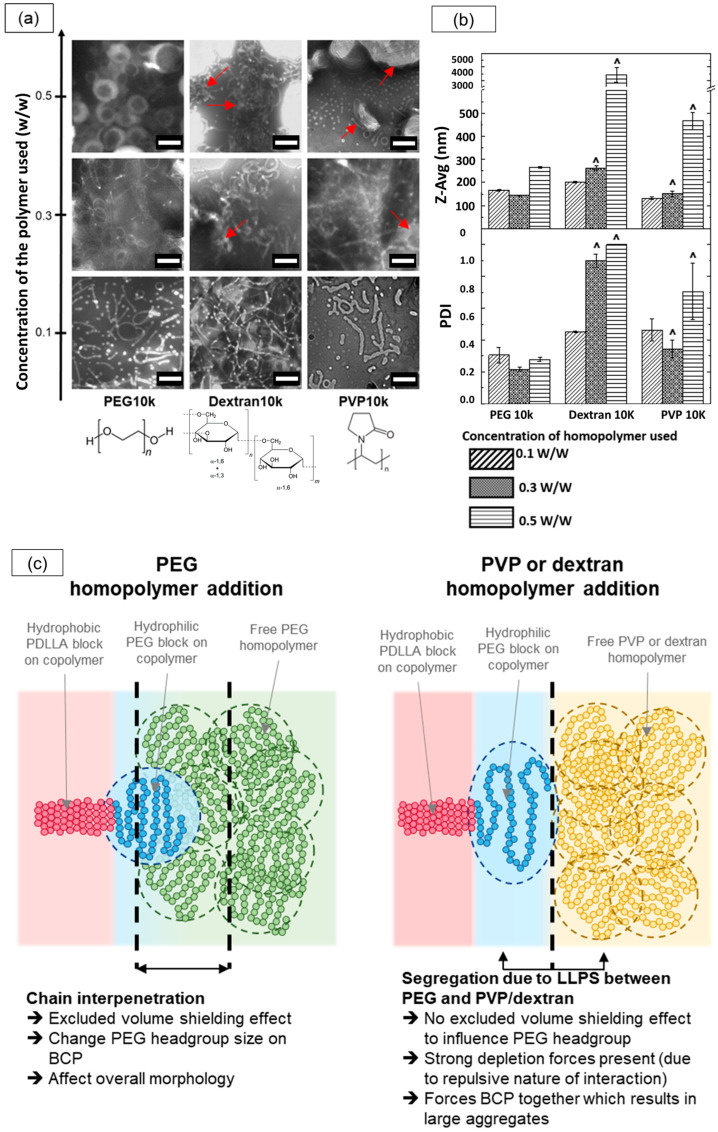
Effect of different homopolymer additives (PEG10k, Dextran10k, and PVP10k) at various concentrations on PEG-PLA nanostructures. (**a**) TEM images of PEG-PLA structures formed with each homopolymer at increasing concentrations, with red arrows indicating large aggregates. Note that due to the limited TEM field of view, larger aggregates may not be fully captured in a single image. Scale bar: 200 nm (**b**) DLS measurements comparing the Z-average size (Z-Avg) and polydispersity index (PDI) of PEG-PLA structures. Note: The symbol “^” denotes samples flagged by the DLS software as too polydisperse, suggesting that measurements may be unreliable. (**c**) Schematic illustrating the potential influence of different homopolymer additives on the self-assembly of PEG-PLA block copolymers, resulting in distinct final nanostructures.

The results from the TEM and dynamic light scattering (DLS) analyses for these assemblies are presented in Figure 8a,b. At lower homopolymer concentrations of 0.3% *w*/*w*, TEM images revealed micellar structures across all three homopolymers, with sub-micron sizes corroborated by the DLS data. However, as the homopolymer concentration increased, the assemblies formed with Dextran10k and PVP10k began to exhibit macroscopic structures, as reflected by larger size distributions in the DLS data. TEM confirmed this shift, showing large, irregular aggregates (highlighted by red arrows), along with some elongated micellar structures. This mixed morphology likely contributed to the high polydispersity index (~1) observed in DLS measurements. In contrast, the PEG10k samples demonstrated a distinct shape transition from micelles to vesicles with increased homopolymer concentrations, while maintaining sub-micron sizes according to DLS.

We attribute this divergence to differences in the interactions of PVP and dextran with the PEG-PLA BCPs compared with PEG. Dextran and PVP are known to undergo macroscopic liquid–liquid phase separation (LLPS) with PEG at sufficiently high concentrations, segregating into two phases, each enriched with its respective homopolymer. In our PEG-PLA system, this segregation effect likely manifests between the PEG block and the added dextran or PVP homopolymers, inhibiting interaction and chain interpenetration between PEG and dextran or PEG and PVP. Consequently, the high dextran or PVP concentrations enhance the depletion of attraction forces, leading to the formation of large aggregates. In contrast, PEG homopolymers are fully compatible with the PEG block on the copolymer, allowing PEG chain interpenetration and promoting excluded volume screening effects that guide the final self-assembly of PEG-PLA into distinct nanostructures. A schematic illustrating these interactions and their effects on morphology is provided in Figure 8c. This study suggests that the compatibility and excluded volume screening effects between the homopolymer additive and PEG-PLA BCPs are critical factors in facilitating morphological transitions in PEG-PLA assemblies.

### 3.7. Thermoresponsive Cargo Delivery Systems

**Figure 9:**
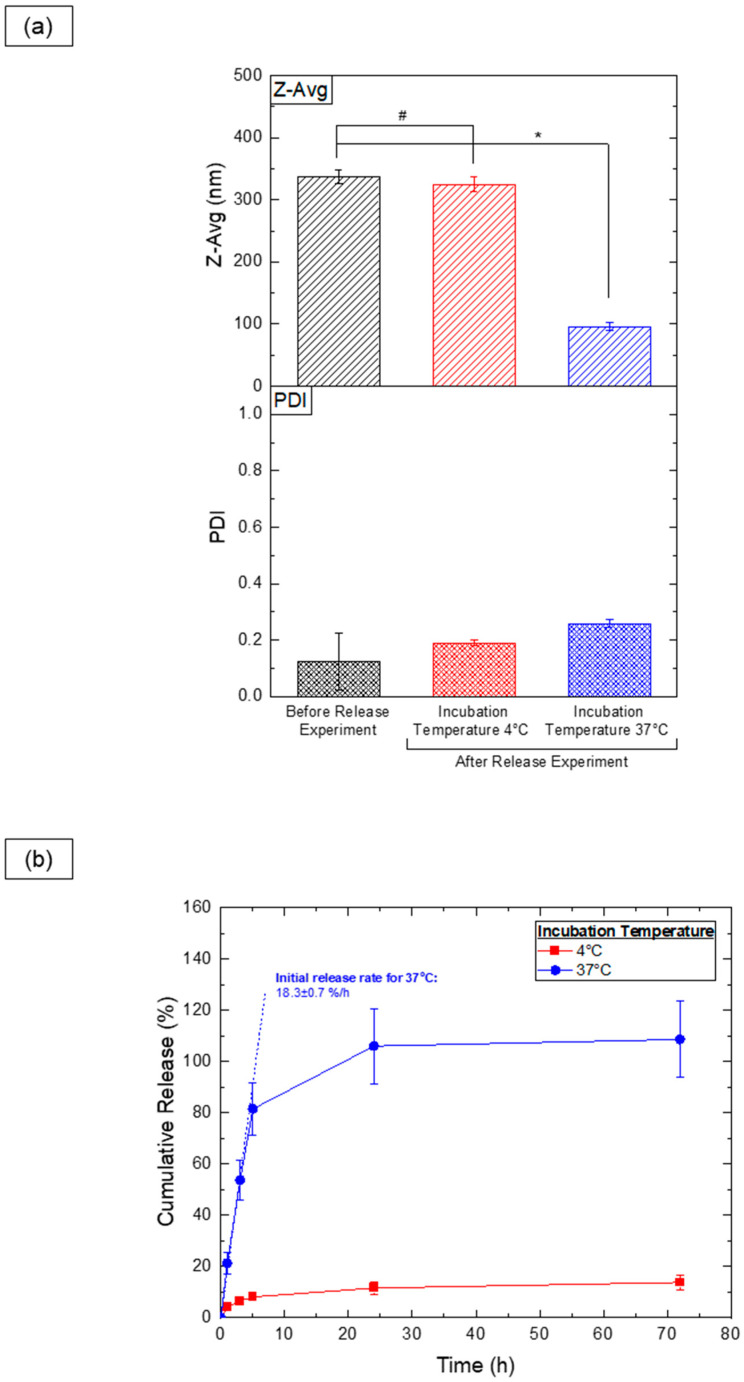
Results of the release experiment for PEG-PLA vesicles incubated at different temperatures. (**a**) DLS analysis of the Z-average (Z-Avg) and polydispersity index (PDI) for samples before and after a 72 h release experiment. Statistically significant differences (*p* ≤ 0.05) are indicated by *, and datasets without statistically significant differences (*p* > 0.05) are marked with #. (**b**) Release profile of NaFluo dye from PEG-PLA vesicles, with the initial release rate calculated from data collected during the first 3 h.

From an application standpoint, PEG-PLA vesicles show promise as thermoresponsive cargo delivery systems. To assess this, vesicles were loaded with hydrophilic sodium fluorescein (NaFluo) dye, and its release was evaluated at both 4 °C and 37 °C. DLS measurements (Figure 9a) revealed significant size changes exclusively in the samples kept at 37 °C, indicating a shape transition following the release experiment. Samples stored at 4 °C showed no notable size variation, aligning with previous findings that shape transitions occur only above the glass transition temperature of PLA. This suggests that vesicles can be stored at 4 °C without significant morphological changes.

Dye release analysis (Figure 9b) demonstrated a substantial NaFluo release at 37 °C, likely due to the vesicle-to-micelle transition, which facilitates the release of dye from within the vesicle’s aqueous cavity. Samples stored at 4 °C retained the majority of the encapsulated dye, with minimal leakage (approximately 10%), possibly due to incomplete encapsulation during the fabrication process. These findings highlight the vesicles’ potential for controlled, temperature-sensitive release in practical applications.

## 4. Discussion

In this work, we investigated the role of PEG homopolymer additives and the kinetic traps provided by the PLA block in the BCPs for controlling the morphology of PEG-PLA nanoparticles. Our results reveal that PEG homopolymers significantly affect the self-assembly of PEG-PLA, inducing a reversible micelle-to-vesicle transition through changes in the concentration and molecular weight. Higher PEG concentrations disrupt the packing of PEG-PLA chains, driving the reorganization from micelles to vesicles. This transformation likely stems from molecular crowding effects at elevated PEG concentrations, with vesicles providing a more stable configuration under such conditions. The transition appears to be predominantly influenced by excluded volume screening effects. At lower PEG concentrations, the PEG head group maintains an expanded conformation that stabilizes micelles [27,28]. As the concentration increases, chain overlap screens these interactions, reducing the PEG coil size and shifting the packing geometry from conical to cylindrical, which promotes vesicle formation. Small-angle neutron scattering (SANS) analysis confirms this shift [30]. Larger PEG chains, such as PEG10k, induce the transition at lower concentrations due to their greater steric hindrance, destabilizing micelles more effectively than shorter chains. These findings align with previous research on homopolymer-induced structural reorganization in BCP systems. Secondary effects, such as interfacial and depletion influences, also contribute to this transition. The lower polarity of PEG relative to water reduces the interfacial curvature at the hydrophilic–hydrophobic boundary, favoring vesicle formation. This effect is more pronounced with lower molecular weight PEG, as higher molecular weight chains may have limited penetration into the micelles’ corona [22]. Depletion attractions caused by non-adsorbing PEG homopolymers can increase micelle aggregation and stretch the hydrophobic PLA block, aiding the transition. However, depletion effects alone do not fully explain the observed transitions, as other homopolymer additives did not replicate these outcomes [31,32]. The temperature dependence further supports the reversible nature of the shape transition. At 4 °C, vesicular structures remain stable, whereas higher temperatures (37 °C and 60 °C) favor a reverse vesicle-to-micelle transition, with higher temperatures accelerating this process. The rapid drop in turbidity at 50 °C indicates a swift transition, while a more gradual decrease at 40 °C suggests a slower transformation. The initial step-like reduction in absorbance at 40 °C and 50 °C reflects a pronounced size decrease early in the transition process, highlighting temperature as a critical factor in the PEG-PLA assembly’s dynamics [33]. These findings corroborate previous studies linking changes in turbidity with particle size, demonstrating the utility of real-time absorbance monitoring at 600 nm for tracking dynamic transitions [34,35]. The dissociation pathway suggests that the system minimizes energetically unfavorable bilayer structures. Removing the PEG homopolymer reduces excluded volume screening, allowing the PEG block to adopt an extended conformation and shifting the packing geometry from cylindrical to conical, favoring micelle formation. Initial pore formation in vesicles likely reflects efforts to create micellar edges with a higher curvature. This transformation resembles the shape changes reported in polymerization-induced self-assembly (PISA) studies, where increasing the hydrophobic-to-hydrophilic BCP ratio drives micelle-to-vesicle transitions [36,37]. Unlike PISA, which requires synthetic modifications and solvents, our method achieves similar polymorphism through straightforward manipulation of the PEG homopolymers’ concentration and molecular weight, offering a robust approach for controlling the BCPs’ morphology. The micelle-to-vesicle transition in PEG-PLA self-assemblies is predominantly driven by excluded volume screening, supported by secondary interfacial and depletion effects. Understanding these mechanisms provides a basis for tailoring BCPs’ morphology for applications such as drug delivery, nanoreactors, and nanomaterial-based technologies.

In conclusion, this study presents experimental evidence demonstrating the influence of non-adsorbing homopolymers at high concentrations on the morphology of self-assembled BCP nanoparticles in solution. This approach enables the formation of diverse nanoparticle shapes from a single BCP without altering the solvophobic-to-solvophilic block ratios. This versatility expands the potential applications of BCP nanoparticles across various fields [38,39]. The insights gained here enhance the understanding of self-assembly in complex systems, with implications for industrial, consumer, and pharmaceutical formulations containing polymeric co-solutes [40,41,42]. Additionally, these findings may extend to biological systems, where macromolecular crowding impacts the behavior of self-assembled structures [43,44], potentially influencing biological outcomes. The observed reverse vesicle-to-micelle transition in PEG-PLA systems offers a promising strategy for designing stimulus-responsive particles capable of adapting to external conditions. Such particles are valuable for targeted delivery systems, offering improved control over the release of active cargo [45,46]. For example, thermoresponsive vesicles developed using PEG-poly(N-isopropylacrylamide) (PEG-PNIPAAM) exhibit vesicle dissociation at 25 °C to facilitate doxorubicin release [12,47]. Similarly, Cerritelli et al. created PEG-disulfide-poly(propylene sulfide) vesicles that disassemble in reducing environments [48]. A key challenge in current research lies in developing BCPs with stimulus-responsive properties, which often requires complex polymer synthesis. Our approach bypasses this challenge by stabilizing self-assembled structures in non-equilibrium states, where external stimuli induce shape transitions. This method enables stimulus-responsive behavior in otherwise unresponsive BCPs like PEG-PLA, broadening the material options for smart nanoparticle systems and simplifying downstream applications.

## 5. Conclusions

In this study, we examined how the interplay between homopolymer additives and kinetic traps influences the morphology and behavior of self-assembling block copolymers (BCPs) in solution, using a system composed of PEG-PLA BCPs and PEG homopolymers in water. Our findings reveal a novel and versatile approach that researchers can use to modulate the morphology of self-assembling BCP nanoparticles. This work highlights the importance of understanding BCP self-assembly in the context of interactions between multiple system components. Such insights can empower scientists to design more effective and complex systems, potentially positioning BCP self-assemblies as a valuable platform for developing next-generation materials with diverse applications.

## Data Availability

Data are contained within the article and Supplementary Materials.

## Supplementary Materials

The following supporting information can be downloaded at https://www.mdpi.com/article/10.3390/nano14242015/s1, Figure S1: Transmission electron microscopy (TEM) images of PEG-PLA structures formed in deionized water with varying PEG molecular weights and concentrations.
