# Supplementary material for: Controlling the Morphology of Poly(ethylene glycol)-b-poly(lactide) Self-Assemblies in Solution: Interplay of Homopolymer Additives and Kinetic Traps
Source: Nanomaterials (Basel). 2024 Dec 14;14(24):2015. doi: 10.3390/nano14242015 (PMC11678193; doi:10.3390/nano14242015)
Supplement: Supplementary file 1 [file nanomaterials-14-02015-s001.zip › nanomaterials-3323374-supplementary.pdf]

Supplementary Information

Controlling the Morphology of Poly(ethylene glycol)-b-poly(lactide) Self-Assemblies in Solution: Interplay of Homopolymer Additives and Kinetic Traps

Pei Qi Lim [1], Srirangam Ramanujam Vaibavi [1], Atul N. Parikh [2], Subbu Venkatraman [3] and Bertrand Czarny [1,4,*]

[1] School of Materials Science & Engineering, Nanyang Technological University, Singapore 639798, Singapore
[2] Departments of Biomedical Engineering and Materials Science & Engineering, University of California, Davis, Davis, CA 95616, USA
[3] Material Science & Engineering, National University of Singapore, Singapore 117546, Singapore
[4] Lee Kong Chain School of Medicine, Nanyang Technological University, Singapore 636921, Singapore
* Correspondence: bczarny@ntu.edu.sg

Supplementary Figures

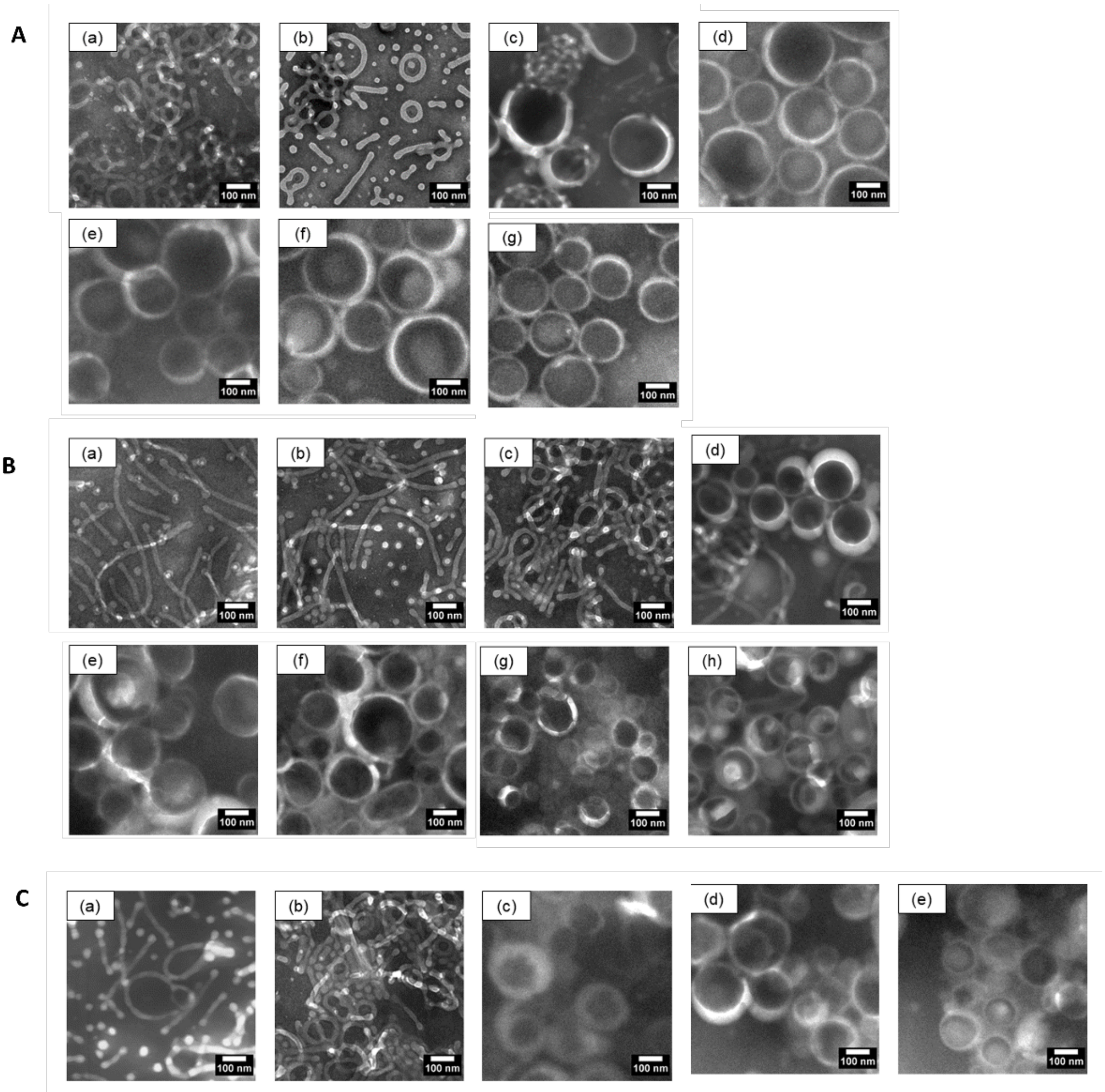

Figure S1: Transmission electron microscopy (TEM) images of PEG-PLA structures formed in deionized water with varying PEG molecular weights and concentrations.
***A** shows PEG2k dissolved in deionized water at varying concentrations: (a) 0.5% w/w, (b) 0.7% w/w, (c) 1.0% w/w, (d) 1.2% w/w, (e) 1.5% w/w, (f) 1.7% w/w, and (g) 2.0% w/w.*
***B** shows PEG6k dissolved in deionized water at varying concentrations: (a) pure deionized water or 0 w/w, (b) 0.1 w/w, (c) 0.3 w/w, (d) 0.5 w/w, (e) 0.7 w/w, (f) 1 w/w, (g) 1.2 w/w, and (h) 1.5 w/w.*
***C** shows PEG10k dissolved in deionized water at varying concentrations: (a) 0.1 w/w, (b) 0.3 w/w, (c) 0.5 w/w, (d) 0.7 w/w, and (e) 1 w/w. Scale bars are indicated on individual panels.*

For PEG2k at lower concentrations (0.5–1.2% w/w), the structures appear as small, dispersed micellar assemblies. As the concentration increases (1.5–2.0% w/w), slight aggregation is observed, indicating a concentration threshold for achieving stable morphology (Figure 1A). For PEG6k, initial formations at low concentrations (0–0.5% w/w) resemble micelles but exhibit greater structural uniformity compared to PEG2k. Transition to vesicle-like structures becomes evident at higher concentrations (0.7–1.5% w/w), driven by molecular weight-dependent chain extension and packing effects (Figure 1B). In contrast, PEG10k demonstrates a pronounced ability to form vesicles even at lower concentrations (0.1–0.5% w/w), suggesting enhanced chain flexibility and compatibility.

At higher concentrations (0.7–1.0% w/w), these structures stabilize into well-defined vesicles with uniform size and shape (Figure 1C).

Supplementary Information:

Experimental Methods:

Materials:

Polyvinylpyrrolidone (PVP) and dextran, both with molecular weights of 10,000 g/mol, were purchased from Sigma-Aldrich and will be referred to as PVP10k and Dextran10k, respectively. These polymers were used as received, without further purification.

Fabrication of Block Copolymer (BCP) Self-Assembled Structures:

The PEG-PLA structures were fabricated following the protocol detailed in the main text. Aqueous solutions containing PEG10k, PVP10k, and Dextran10k were prepared at concentrations of 0.1% w/w, 0.3% w/w, and 0.5% w/w.

Dynamic Light Scattering (DLS) Characterization:

Dynamic light scattering (Nano-ZS, Malvern) was used to determine the intensity-weighted mean diameter (Z-average) and polydispersity index (PDI) of the samples. For each measurement, samples were diluted to approximately 0.25 mg/mL in deionized water and loaded into pre-rinsed cuvettes. All measurements were performed at room temperature.

Differential Scanning Calorimetry (DSC) Characterization:

Differential scanning calorimetry (Nano DSC, TA Instruments) was used to analyze the PEG-PLA samples. The samples were loaded directly into the sample cell, while the reference cell was filled with deionized water. The sample chamber was pressurized to 3 atm, and a thermal scan was conducted at a heating rate of 1°C per minute.
